# Circulating brain-derived extracellular vesicles expressing neuroinflammatory markers are associated with HIV-related neurocognitive impairment

**DOI:** 10.3389/fimmu.2022.1033712

**Published:** 2022-12-19

**Authors:** Erika G. Marques de Menezes, Jocelyn S. Liu, Scott A. Bowler, Leila B. Giron, Michelle L. D’Antoni, Cecilia M. Shikuma, Mohamed Abdel-Mohsen, Lishomwa C. Ndhlovu, Philip J. Norris

**Affiliations:** ^1^Vitalant Research Institute, San Francisco, CA, United States; ^2^Department of Laboratory Medicine, University of California, San Francisco, CA, United States; ^3^Division of Infectious Diseases, Department of Medicine, Weill Cornell Medicine, New York, NY, United States; ^4^The Wistar Institute, Philadelphia, PA, United States; ^5^Department of Tropical Medicine, John A. Burns School of Medicine, University of Hawaii, Honolulu, HI, United States; ^6^Hawaii Center for AIDS, John A. Burns School of Medicine, University of Hawaii, Honolulu, HI, United States; ^7^Department of Medicine, University of California, San Francisco, CA, United States

**Keywords:** extracellular vesicles, monocytes, neurons, neurocognitive impairment, human immunodeficiency virus

## Abstract

**Background:**

Neurocognitive impairment remains prevalent in people with HIV (PWH) despite long term virological suppression by antiretroviral therapy (ART) regimens. Systemic and neuro-inflammatory processes are suggested to contribute to the complex pathology leading to cognitive impairment in this population, yet the underlying mechanisms remain unresolved. Extracellular vesicles (EVs) play a central role in intracellular communication and have emerged as key modulators of immunological and inflammatory responses. In this report, we examined the impact of EVs in PWH experiencing cognitive deficits to determine their relevance in HIV associated neuropathology.

**Methods:**

EV phenotypes were measured in plasma samples from 108 PWH with either cognitive impairment (CI, n=92) or normal cognition (NC, n=16) by flow cytometry. Matched cerebrospinal fluid (CSF)-derived EVs were similarly profiled from a subgroup of 84 individuals who underwent a lumbar puncture. Peripheral blood mononuclear cells were assayed by flow cytometry to measure monocyte frequencies in a subset of 32 individuals.

**Results:**

Plasma-EVs expressing CD14, CD16, CD192, C195, and GFAP were significantly higher in HIV-infected individuals with cognitive impairment compared to individuals with normal cognition. Increased CSF-EVs expressing GFAP and CD200 were found in the cognitive impairment group compared to the normal cognition group. Frequencies of patrolling monocytes correlated with plasma-EVs expressing CD14, CD66b, MCSF, MAP2, and GFAP. Frequencies of CD195 expression on monocytes correlated positively with plasma-EVs expressing CD41a, CD62P, and CD63. Expression of CD163 on monocytes correlated positively with CSF-EVs expressing GFAP and CD200. Finally, the expression of CD192 on total monocytes correlated with CSF-EVs expressing CD200, CD62P, and CD63.

**Conclusions:**

EVs expressing monocyte activation and neuronal markers associated with HIV associated cognitive impairment, suggesting that distinct EV subsets may serve as novel biomarkers of neuronal injury in HIV infection. Further circulating platelet EV levels were linked to monocyte activation indicating a potential novel interaction in the pathogenesis of HIV-related cognitive impairment.

## Introduction

Despite effective antiretroviral therapy (ART), people with HIV (PWH) experience a state of chronic low-level inflammation and immune activation that may contribute to the pathogenesis of HIV-associated neurocognitive (HAND) (1–3). In the ART era the incidence and severity of HIV-associated dementia (HAD) have decreased; however, asymptomatic neurocognitive impairment (ANI) and minor cognitive motor disorder (MCMD) persist in 10-30% of PWH (4–6). These cognitive deficits are not only widespread but also can affect everyday functioning and increase morbidity and mortality (7–9). While the migration of activated and infected monocytes across the blood-brain barrier, drugs of abuse, the secondary effects of aging, and persistent viral replication may be contributing factors to neurological damage in the central nervous systems (CNS) (10, 11), it has been challenging to identify molecular mechanisms that can be targeted to reduce neuroinflammation. In addition, noninvasive biomarkers for neurocognitive disorders in HIV infection is still needed, not only for diagnosis but to allow monitoring of potential disease interventions.

Extracellular vesicles (EVs) including exosomes and microvesicles, are membrane-bound particles of cellular origin involved in regulating many pathophysiological and normal functions in the body, including immune responses, inflammation, and cell death (12–14). As a messenger of intercellular communication, EVs contain a rich cargo of proteins, nucleic acids, lipids, and diverse molecules (15–17). They are shed by most cell types and are found in cell-culture media and body fluids, including blood and cerebrospinal fluid (CSF) (18–20). Evidence indicates that EVs play a key role in the activity of the nervous system, providing a mechanism of intercellular communication between the CNS with other body systems (21, 22). Furthermore, EVs can move across the blood-brain barrier and deliver biological materials to cells in the brain, making them a promising new avenue of investigation for CNS functioning and for the identification of new biomarkers for neurodegenerative diseases (23–25). In addition, we recently showed that plasma-EVs expressing monocyte markers are associated with carotid artery intima-media thickness in HIV-infected individuals on virologically suppressive ART. We further showed that the EV fraction from HIV+ adults on stable ART induced endothelial cell death *via* necrosis of human umbilical vein endothelial cells (26). These properties make EVs potential candidates as targets of immunotherapies and putative biomarkers for diseases (17, 27–30).

To investigate the relevance of how EVs deliver immune signals in the setting of HIV, both in the periphery and the CNS, we examined whether levels of circulating EVs expressing monocyte inflammatory phenotypic and markers of neuronal damage differed between HIV-infected individuals with or without CI. We also determined whether circulating monocytes subsets associated with EVs expressing surface markers spanning lymphoid, myeloid, and neurological cell lineages.

## Methods

### Study participants

The Hawaii Aging with HIV Cohort (HAHC) study is a longitudinal cohort established and designed to study the impact of age and HIV on cognitive function (31). Medical and ART history, neuropsychological testing, demographic information, and clinical laboratory assessments were collected. Stored plasma, peripheral blood mononuclear cells (PBMCs), and CSF from this cohort were cryopreserved. Exclusion criteria included if the participants had previously been diagnosed with an opportunistic infection, neoplasia, hepatic impairment, head injury, learning disability, major neurologic/psychiatric disorder, opportunistic brain infection, or active substance abuse. This study was approved by the Institutional Review Board of the University of Hawaii Committee on Human Subjects, and all participants gave written informed consent.

For the quantitation of EV surface markers, a total of 108 plasma samples were collected from HIV-infected individuals, having either normal cognition (n=16) or cognitive impairment based on the American Academy of Neurology (AAN) criteria for HIV-associated dementia (HAD, n=25), minor cognitive motor disorder (MCMD, n=38) or asymptomatic neurocognitive impairment (ANI, n=29) (32). Matched CSF-derived EVs were profiled from a subgroup of 84 individuals with normal cognition (n=10) or with cognitive impairment (HAD, n=19; MCMD, n=33; ANI, n=22), and PBMCs were assayed by flow cytometry to measure monocyte frequencies in a subgroup of 32 individuals with normal cognition (n=3) or cognitive impairment (HAD, n=12; MCMD, n=12; abnormal, n=5).

### Neurocognitive assessment

Neuropsychological assessments were performed by using a comprehensive battery of eight tests (NPZ-8 score) and additional assessments included the macro-neurologic examination, medical and medication histories, risk behavior inventory, and neurocognitive testing. The NPZ-8 battery tests assessed multiple cognitive domains and included the following: grooved pegboard, trail making tests A and B, timed gait, digit symbol test, odd man out, animal naming, Boston naming test, basic choice and sequential reaction time, and Rey auditory verbal learning test (31, 33). In addition, raw scores were transformed to *z*-scores and subdomain scores were calculated as previously described (34). To better discriminate neuropsychological subset sensitivity in our analysis, we also used the geriatric depression scale (GDS), a self-reported measure of symptoms of depression (35, 36). Additional tests were included to address working memory, auditory span of attention, verbal fluency, and execute functions as previously described (37–39). All neuropsychological testing was performed by an examiner trained and supervised by a clinical neuropsychologist.

### Biological sample EV processing

CSF samples were obtained by lumbar puncture in the L3/L4 or L4/L5 intervertebral space using a 25-gauge needle and were collected in sterile polypropylene tubes. Samples were centrifuged at 2000 g for 10 minutes at 4°C, and stored at -80°C. Briefly, samples were thawed and immediately processed in order to limit the freeze-thaw cycles. The samples were centrifuged at 2000 g and referred to as large EVs as previously reported in an ISEV position paper (40) and used to measure EV concentration and phenotype.

**Figure 1:**
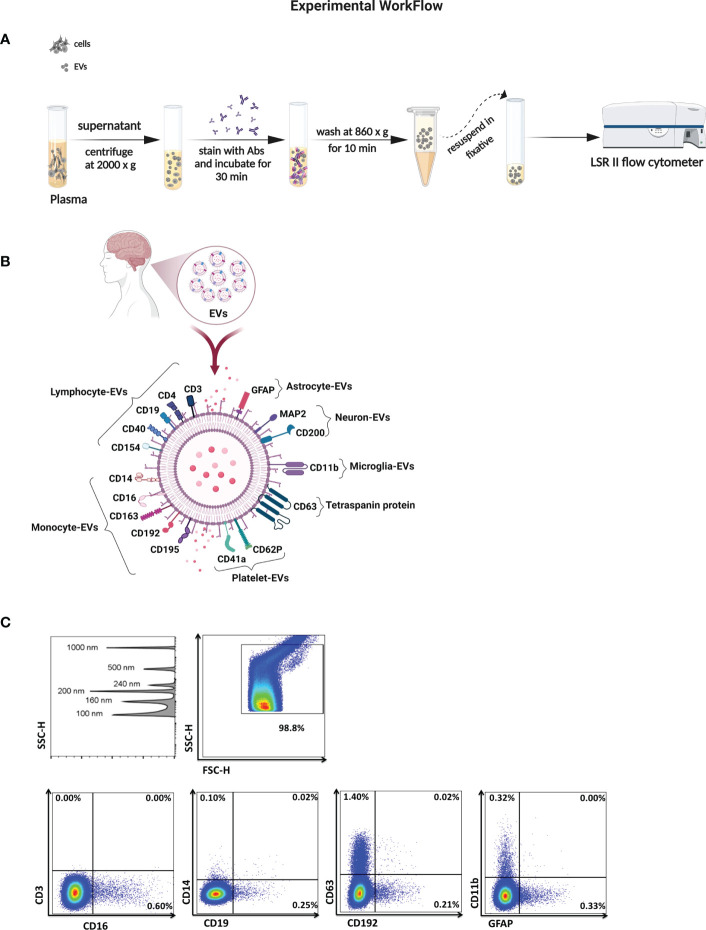
Characterization of EVs in biological samples. **(A)** Schematic of the method for isolation and analysis of EVs for their absolute count and cell of origin. **(B)** Illustration of surface markers on EVs linked to neuronal cells, lymphoid cells, and myleoid cells. **(C)** SSC height (SSC-H) dot plot shows sensitivity to detect beads sized 100 to 1000 nm in diameter. Representative plots of EV gated from the threshold to the 1000 nm gate based on SSC and sorted according to surface markers from their cell of origin.

Fasting morning blood samples were collected into EDTA tubes, centrifuged at 2000 g for 10 minutes at 4°C, and the obtained plasma was stored at -80°C until further analysis. Briefly, the supernatant was thawed and centrifuged through 0.22 µm centrifugal filter (Millipore) for 10 minutes at 860 g or until most supernatant had passed through. EVs were resuspended in 500 µL of filtered PBS containing 2.8% formaldehyde (BD stabilizing fixative), and their concentration and phenotype were measured using a previously described approach (**Figure 1A**) (41, 42).

### EV immunophenotyping by flow cytometry

To measure EV numbers and expression of surface markers, blinded plasma and CSF samples were stained using pre-titrated volumes of the following fluorochrome-conjugated monoclonal antibodies in four separate panels, listed here according to their cell of origin and purchased from BioLegend unless otherwise noted: lymphocytes CD3 (Clone: OK-T3), CD4-PE (SK3), and CD19-PECy7 (SJ25CI, BD Biosciences), CD40-FITC (5C3), and CD154-APC (2431), monocytes CD14-APC (63D3), CD16-V421 (3G8), CD163-PECy7 (GHI6I), CD192-V421 (K036C2) CCR2, and CD195-PE (2D7) CCR5, BD Biosciences), macrophage colony-stimulating factor (MCSF)-PE (26786), granulocytes CD66b-PerCP/Cy5.5 (G10FS), platelets CD41a-PerCP/Cy5.5 (HIP8), and CD62P-FITC (AK-4 BD Biosciences), tetraspanin protein CD63-APC (H5C6), astrocytes GFAP-V421 (glial fibrillary acidic protein (2E1E9), microglia CD11b-PECy7 (OKT3), neurons CD200-PerCP/Cy5.5 (OX104) and microtubule-associated proteins (MAP2)-Alexa 488 (18MAP2B, BD Biosciences) (**Figure 1B**). Briefly, when panels were optimized, fluorescence minus one controls were used to determine the level of background fluorescence. Prior to testing EV samples, antibody filtration was performed using a pore size of 0.22 µm centrifugal filter, and the filtrate was used for staining. One to 5 µL of titrated monoclonal antibodies was added to 10 µL of plasma-EVs and to 100 µL of CSF-EVs and incubated at 4°C for 30 minutes. EVs were diluted in buffered 0.22 µm-filtered PBS containing 2.8% formaldehyde (BD stabilizing fixative) to appropriate dilutions to prevent coincident detection, and each EV sample was run simultaneously with an unstained sample.

Acquisition was performed on an LSRII flow cytometer (Becton Dickinson). Forward scatter (FSC) and side scatter (SSC) were set to the voltages around of 500-600 and 300-400, respectively. The side scatter was set to a triggering threshold of 200arbitrary units. A 0.22 µm-filtered PBS control was recorded to estimate the background signal. EV gates were established 100 nm (Invitrogen) to 1000 nm beads (Megamix: 160, 200, 240 and 500 nm; Spherotech 1000 nm) (26, 41, 42). Representative flow cytometry plots are shown in **Figure 1C**. Samples were acquired for 60 seconds at a low flow rate (8 – 12 µL/min), and the concentration of EVs was calculated using TruCount™ tubes (BD Biosciences). Analysis was performed using FlowJo 10.7.1 software (BD).

### PBMC flow cytometry phenotyping

Cryopreserved PBMCs were thawed and stained for 30 minutes at 4°C with viability dye (yellow Live/Dead Fixable Dead Cell stain, Invitrogen), followed by staining with monoclonal antibodies against CD14, CD16, CD162, CD163, CD192, CD195, CX3CR1, and HLA-DR. All antibodies were from BD Biosciences. Control studies with unstained cells and cells incubated with appropriate isotype-matched for each antibody were used as a negative control. In the FSC-SSC dot plot, a biparametric gate was drawn around the monocyte population as previously described (43). Monocytes, positive for HLA-DR were then classified into three subsets based on the expression of CD14, CD16markers: classical (CD14^++^CD16^-^), intermediate (CD14^++^CD16^+^), or non-classical (CD14^+^CD16^++^) monocytes. Monocytes populations were further assessed for CD162, CD163, CD192, CD195, and CX3CR1 expression. Cells were fixed with 1% formaldehyde solution (BD Biosciences) and measured on a four-laser custom BD-Fortessa flow cytometer (Becton Dickinson). Compensation and gating analyses were performed using FlowJo software (BD).

### Statistical analysis

Statistical analyses were performed using Prism 7.0 (GraphPad Software). Subject demographic and clinical characteristics between the normal cognition and cognitive impairment subgroups (ANI, MCMD, and HAD) were compared by Kruskal-Wallis test with Dunn’s Multiple Comparison *post hoc* test, and those between categorical variables were examined using chi-square test. EV data were log_10_ transformed prior to analysis. The Shapiro-Wilk test was used to determine normality for all datasets prior to implementation of the unpaired *t*-tests. The nonparametric Mann-Whitney U test was used for unpaired comparisons. The Spearman correlation matrix test was used to determine pairwise associations between variables. The data are expressed as means ± standard deviation, unless otherwise indicated. A level of *P* ≤0.05 was considered statistically significant. The p-values are two-sided. The false discovery rate (FDR) for each type of comparison was calculated using the Benjamini and Hochberg where indicated (44), and FDR <0.1 was used as a significance threshold.

## Results

### Demographics and clinical characteristics

Of 108 enrolled HIV-infected participants, over 82% were male, with no significant differences in gender across the NC, ANI, MCMD, and HAD groups. We estimated the duration of viral suppression using the shortest duration of combination ART (cART) regimens, including NRTIs, NNRTIs, and PIs. Integrase inhibitors had not been approved, and no patients were on fusion inhibitors. There were no significant differences in the duration of cART therapy between the NC, ANI, MCMD, and HAD groups (**Table 1A**). The mean age ranged from 44 to 49 years, and there was no significant difference between the groups with respect to age. Significant differences were observed in CD4+ T cell count between the MCMD and HAD groups (p=0.028) and CD4+ T cell count nadir (cells/µL) between the ANI and HAD groups (p=0.017). Of the 108 participants, 55 had detectable plasma HIV RNA, 9 had diabetes mellitus (DM), 8 had hypertension, 4 had prior history of myocardial infarction (MI), 3 had a history of stroke, 43 were current smokers, and 31 had a prior history of smoking. No significant association between the co-morbid states DM, MI, stroke, or current use/history of smoking was observed. However, hypertension was borderline significantly more prevalent in participants with HAD (p=0.050). Detailed demographic and clinical characteristics are presented for the subgroup of 84 HIV-infected individuals for whom matched CSF samples were available (**Table 1B**).

**Table 1A:** Demographic and clinical characteristics of HIV-infected participants.

Participants characteristics	NC *N*= 16	ANI *N*= 29	MCMD *N=* 38	HAD *N*= 25	*P* Value
Demographics					
Age, yrs	44 ± 12	43 ± 11	43 ± 11	49 ± 9	0.165
Male [N (%)]	13 (82)	26 (90)	33 (87)	24 (96)	0.497
BMI (Kg/m^2^)	25 (22 – 27)	24 (22 – 26)	24 (22 – 27)	25 (22 – 27)	0.801
Education (years)	12 (12 – 16)	14 (12 – 16)	14 (12 – 16)	14 (12 – 16)	0.865
NPZ global score*	0.4 (0.1 – 0.5)	-0.1 (-0.7 – 0.2)	-0.5 (-0.9 – 0.1)	-1.1 (-1.7 – -0.5)	<.0001
GDS*	0.7 (0.1 – 0.2)	0.3 (0.2 – 0.7)	0.4 (0.3 – 0.7)	1.1 (0.8 – 1.3)	<.0001
HIV-related					
ART, years^a^	2.1 (0.59 – 5.07)	1.47 (0.77 – 3.08)	1.5 (0.64 – 3.44)	2.75 (1.88 – 4.64)	0.502
CD4+ nadir (cells/µL)	265 (137 – 434)	202 (130 – 500)	180 (53 – 351)	90 (36 – 237)	0.017
CD4+ count (cells/µL)	420 (240 – 619)	426 (306 – 673)	520 (308 – 651)	230 (123 – 610)	0.028
HIV viral load, copies/mL	50 (50 – 2534)	211 (50 – 31,070)	133 (50 – 29,746)	137 (50 – 105,629)	0.711

Results are reported as mean values ± SD or median with interquartile ranges. ^a^Certain data were not available for all participants: NC= 9, ANI= 13, MCMD= 17, and HAD= 11.

*significant differences between the NC group and ANI, MCMD, HAD groups by Kruskal-Wallis test with Dunn´s Multiple Comparison post hoc test. NC, normal cognition; ANI, asymptomatic neurocognitive impairment; MCMD, minor cognitive motor disorder; HAD, HIV-associated dementia; GDS, geriatric depression scale; NPZ global score, neuropsychological z score; ART, antiretroviral therapy.

**Table 1B:** Demographic and clinical characteristics of study participants.

CSF subset	NC *N*= 10	ANI *N*= 22	MCMD *N=* 33	HAD *N*= 19	*P* Value
Demographics					
Age, yrs	39 ± 10	42 ± 11	47 ± 9	48 ± 10	0.128
Male [N (%)]	8 (80)	20 (91)	29 (88)	18 (95)	0.659
BMI (Kg/m^2^)	23 (21 – 26)	23 (21 – 26)	24 (22 – 26)	26 (21 – 29)	0.693
Education (years)	12 (12 – 15)	14 (12 – 16)	14 (12 – 16)	14 (12 – 16)	0.709
NPZ global score*	0.4 (0.2 – 0.5)	-0.1 (-0.8 – 0.2)	-0.5 (-0.9 – 0.1)	-1.0 (-1.4 – -0.5)	<.0001
GDS*	0.02 (0 – 0.1)	0.3 (0.2 – 0.7)	0.4 (0.2 – 0.6)	1.1 (0.8 – 1.2)	<.0001
HIV-related					
CD4+ nadir (cells/µL)	279 (154 – 426)	372 (135 – 530)	168 (28 – 300)	90 (46 – 274)	0.028
CD4+ count (cells/µL)	387 (203 – 601)	437 (306 – 680)	507 (287 – 590)	262 (120 – 645)	0.102
HIV viral load, copies/mL	1238 (50 – 25,051)	1184 (50 – 44,722)	291 (50 – 35,367)	137 (50 – 125,872)	0.985

Results are reported as mean values ± SD or median with interquartile ranges. *significant differences between the NC group and ANI, MCMD, and HAD groups by Kruskal-Wallis test with Dunn´s Multiple Comparison post hoc test. NC, normal cognition; ANI, asymptomatic neurocognitive impairment; MCMD, minor cognitive motor disorder; HAD, HIV-associated dementia; GDS, geriatric depression scale; NPZ global score, neuropsychological z score.

### EVs expressing inflammatory phenotypes are associated with HIV-related neurocognitive impairment

**Figure 2:**
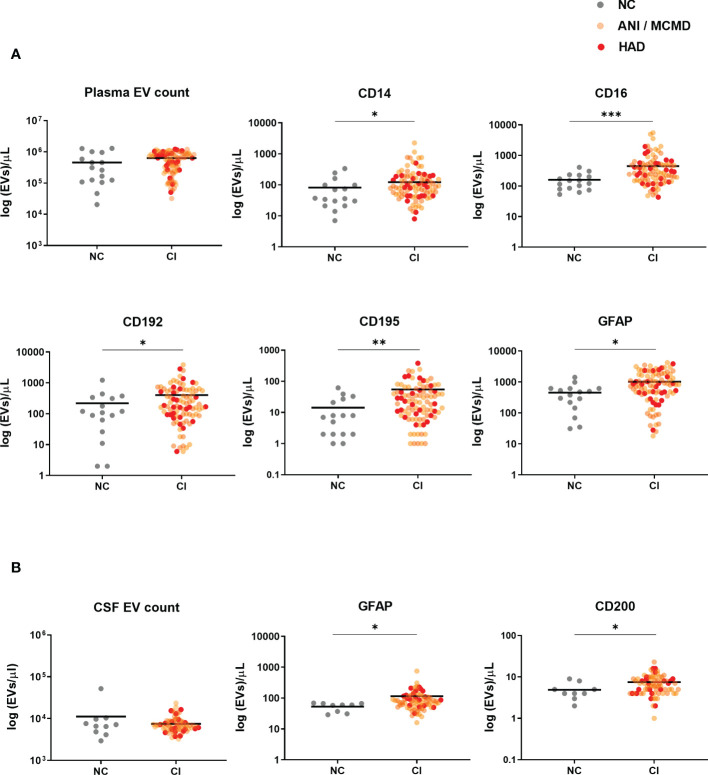
Circulating EVs expressing monocyte-associated markers and neuronal damage associate with cognitive impairment in HIV-infected individuals on stable ART. Scatter plots of EVs/µL numbers (log 10-transformed). **(A)** Plasma-EVs expressing monocyte-associated markers (CD14, CD16, CD192, and CD195) and neuronal damage marker (GFAP) were significantly higher in HIV+ adults with cognitive impairment (CI) compared to those with normal cognition (NC). **(B)** There was a significant increase in levels of CSF-EVs expressing GFAP+ and CD200+ in the CI compared to the NC group. *P* values were determined by using two-tailed Mann-Whitney test. *P<0.05, **P<0.01, ***P<0.001.

To examine whether plasma-derived EVs associate with markers of cellular activation and neuroinflammation in HIV-infected individuals with cognitive impairment, we tested four panels consisting of several antigens linked to neuronal cells (GFAP, MAP2, CD11b, and CD200), lymphoid cells (CD3, CD4, CD19, CD40, and CD154), myeloid cells (CD14, CD16, CD163, CD192, CD195, CD41a, CD62P, CD66b, and MCSF), and multivesicular bodies (CD63). When the quantity of plasma-EVs from HIV-infected individuals with and without cognitive impairment was compared, we found no statistical difference in the concentration of EVs. However, we found significant elevations in levels of EVs expressing monocyte-associated markers in HIV+ persons with cognitive impairment including CD14+EVs, CD16+EVs, CD192+EVs, CD195+EVs, as well as EVs expressing glial fibrillary acidic protein (GFAP) (**Figure 2A**). There were no significant differences in EVs expressing CD3, CD4, CD19, CD11b, CD40, CD41a, CD62P, CD63, CD66b, CD154, CD163, MCSF, CD200, and MAP2 between the cognitive impairment and normal cognition groups (**Figure S1**).

We next assessed whether EV counts and phenotype were elevated in the CSF of HAND individuals. There were no significant differences in the concentration of EVs between the groups. However, we found a significant increase in CSF-EVs expressing two of the neuronal-associated markers, GFAP and CD200, in HIV+ individuals with cognitive impairment group compared to those without cognitive impairment (**Figure 2B**). None of the other markers spanning the lymphoid and myeloid lineages showed differences among study groups. Of note, there was no significant relationship between circulating EVs and plasma viral load (data not shown). These results showed that both CSF and plasma-derived EVs expressing GFAP correlated with cognitive impairment in HIV-infected individuals, with the largest increase seen in plasma-EVs expressing monocyte-associate markers in HIV+ persons with cognitive impairment.

**Figure 3:**
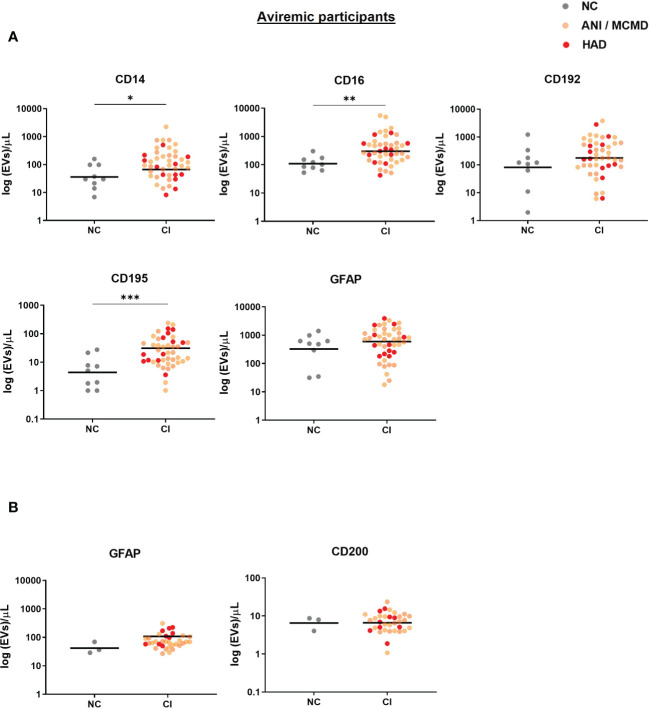
Sensitive analysis of circulating EVs expressing monocyte-associated markers and neuronal damage associate with cognitive impairment in virally-suppressed HIV+ individuals. Scatter plots of total EV numbers and concentration of each subtype of EV are shown. **(A)** levels of EVs expressing CD14, CD16, and CD195 were elevated in the plasma of aviremic HIV-infected individuals with CI compared to the NC group. **(B)** There was no significant difference in levels of GFAP+EVs and CD200+EVs in the CSF of aviremic CI compared to the NC group. *P* values were determined by using two-tailed Mann-Whitney test. *P<0.05, **P<0.01, ***P<0.001. NC, normal cognition; CI, cognitive impairment.

A sensitivity analysis was performed to exclude the viremic individuals (viral load >50 RNA copies/mL), and levels of plasma-EVs expressing monocyte-associated markers remained significantly elevated in the aviremic HIV-infected individual with cognitive impairment (**Figure 3**). Finally, we examined the relationship between all EV subtypes in plasma and CSF based on their surface marker phenotype. Using Spearman’s rank correlation, no significant correlations were found between EV phenotypes and clinical global deficit scores (GDS and NPZ8 score; data not shown).

### EV phenotypes associate with peripheral blood monocytes in individuals with cognitive impairment

**Figure 4:**
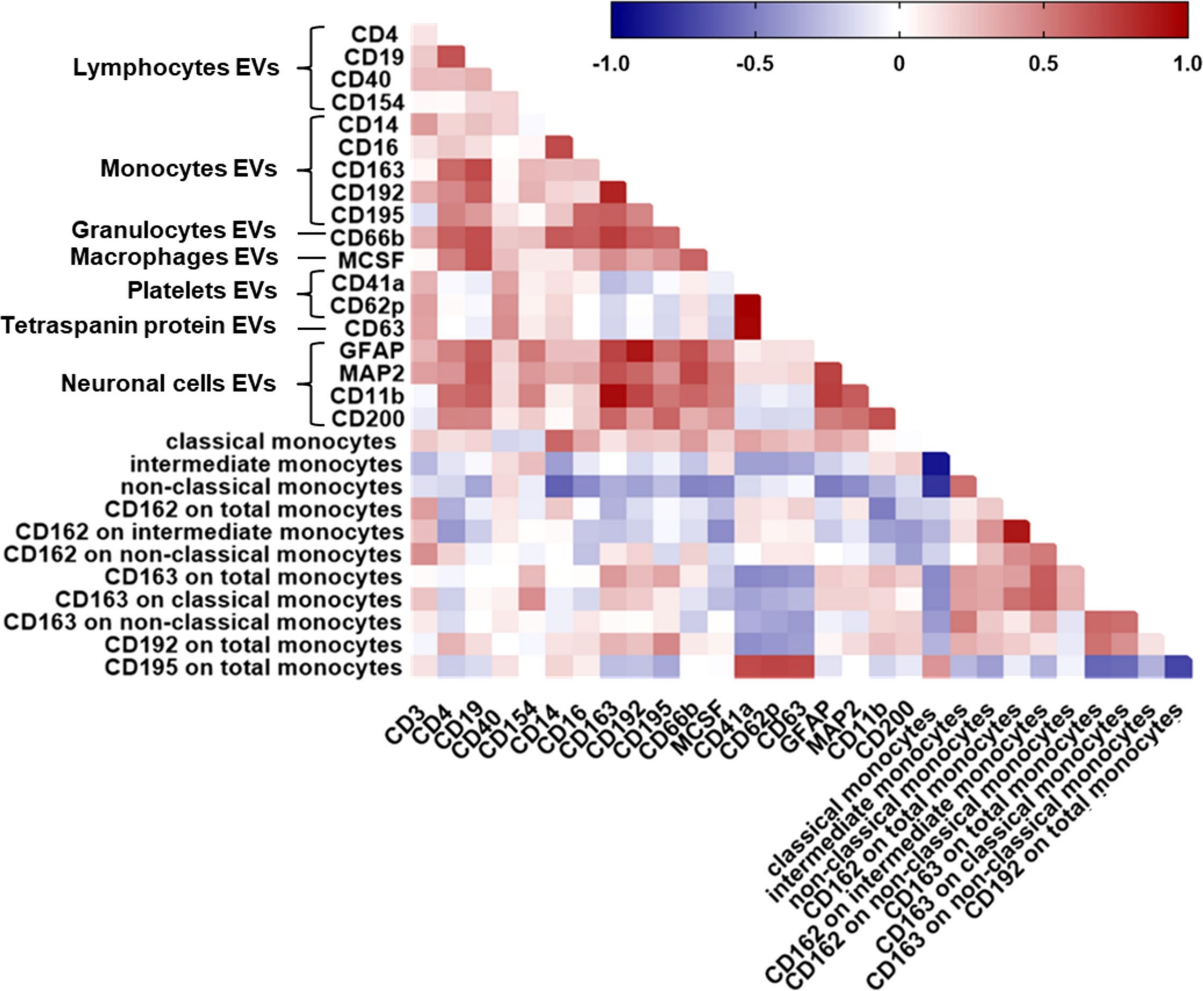
Relationship between all plasma-EV subtypes and circulating monocyte subsets. Spearman correlation matrix showing the association between all EV subtypes based on their cellular origin and circulating monocyte subsets in HIV-infected individuals on ART. Positive correlations are displayed in red shades and negative correlations are in blue shades.

We next analyzed the relationship of PBMC-derived monocytes with EV phenotypes in a subset of 32 HIV-infected individuals for whom both data sets were available. We observed a significant correlation between percent classical monocytes and plasma-EVs expressing CD14 (**Figure 4**). A negative relationship was observed between non-classical monocytes and levels of EVs expressing the monocyte marker CD14, the neutrophil marker CD66b, and the CNS cell markers MAP2, GFAP, and MCSF.

We therefore assessed the relationship of monocyte CD162 (P-selectin glycoprotein ligand 1), CD163 (haptoglobin-hemoglobin scavenger receptor), CD192 (CC chemokine receptor 2), and CD195 (CC chemokine receptor 5) surface expression and plasma-EV inflammatory surface markers due to their known importance as biomarkers for immune activation and/or resolution of inflammation in HIV-infected individuals associated neurocognitive impairment. MFI of CD162 on total monocytes correlated negatively with CD11b+EVs. MFI of CD162 on intermediate monocytes correlated negatively with levels of EVs expressing CD4, and MCSF. There was a negative relationship between the percentage of CD163 on total monocytes and EVs expressing CD41a and CD62P. Negative correlations were also observed between MFI of CD192 on total monocytes and EVs expressing CD41a (**Figure 4**). Frequencies of CD195 on total monocytes correlated positively with CD41a+EVs, CD62P+EVs, and CD63+EVs (**Figure 4**). These associations remained significant after correcting for multiple testing with an FDR of <0.1. Taken together, these results suggest that circulating monocyte subsets are associated with pro-inflammatory EVs, and monocyte activation associated with circulating platelet EV levels, indicating the interaction between platelet activation-associated phenotypes and monocytes in HIV-infected individuals with cognitive impairment.

**Figure 5:**
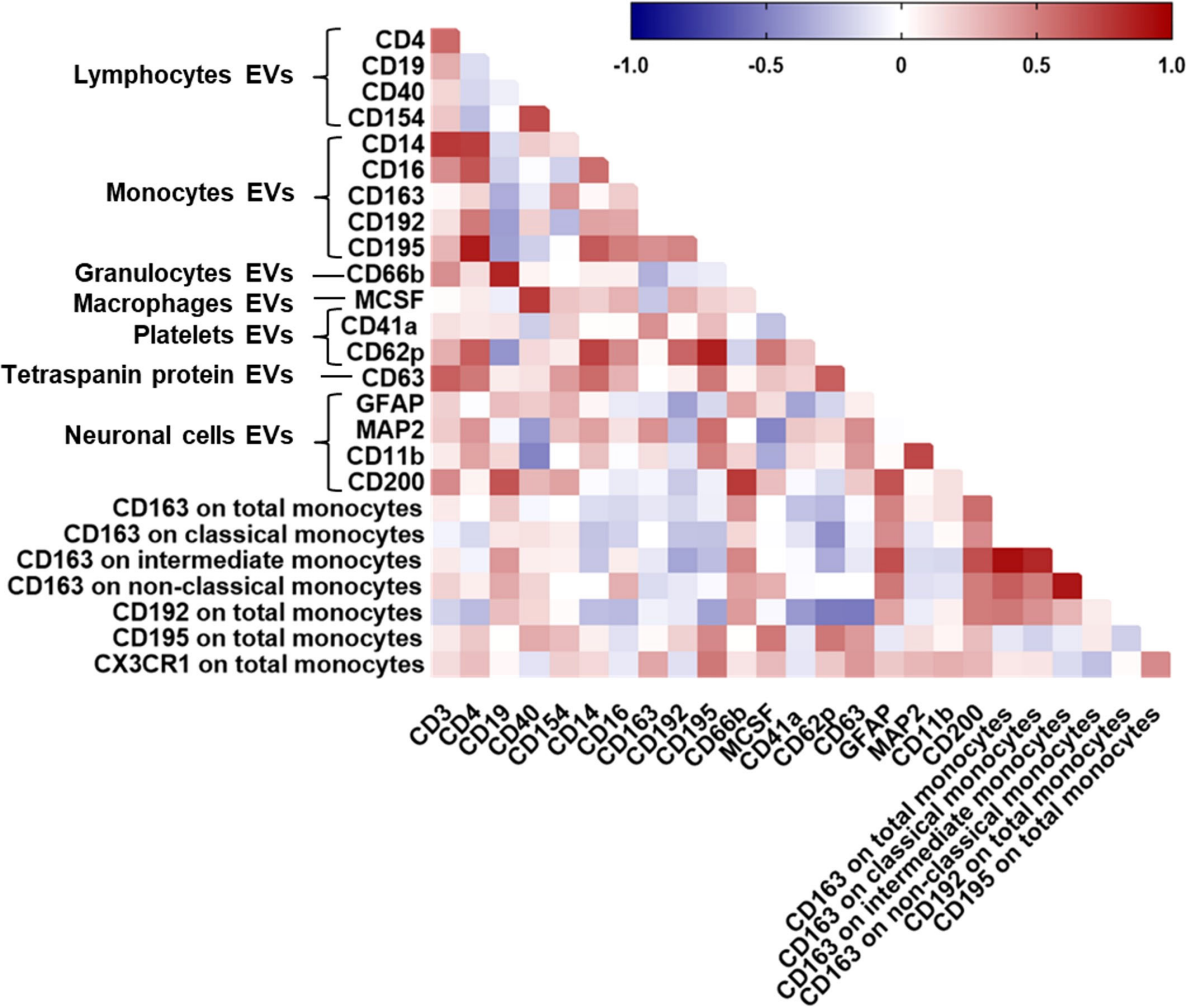
Correlations between all CSF-EV subtypes and circulating monocyte subsets. Spearman correlation matrix showing the association between all CSF-EV subtypes and circulating monocyte subsets in HIV-infected individuals on stable ART. Positive correlations are displayed in red shades and negative correlations are in blue shades.

Finally, to further assess generalizability of the data, we also examined correlations between CSF-EV phenotypes and circulating monocyte subsets in peripheral blood from HIV-infected individuals with cognitive impairment. There was a significant positive correlation between the percentage of CX3CR1 on total monocytes and EVs expressing CD195 (**Figure 5**). MFI of CD192 on total monocytes correlated positively with CD200+EVs and negatively with CD63+EVs and CD62P+EVs. We found a significant positive correlation between the percentage of CD163 on non-classical monocytes and GFAP+EVs and CD200+EVs. We also found a positive correlation between the percentage of CD163 on intermediate monocytes and GFAP+EVs and CD200+EVs. Finally, MFI of CD163 on total monocytes correlated positively with GFAP+EVs and CD200+EVs. These associations remained significant after correcting for multiple comparisons (FDR of <0.1). These data demonstrate correlation between neuronal damage markers in the CSF and monocytes in the periphery in HIV-infected individuals with cognitive impairment.

These results suggest that EVs associate not only with predictors of cognitive impairment but also with peripheral blood monocytes in ART-treated HIV-positive individuals with cognitive impairment, suggesting cross-talk between the periphery and brain during disease.

## Discussion

**Figure 6:**
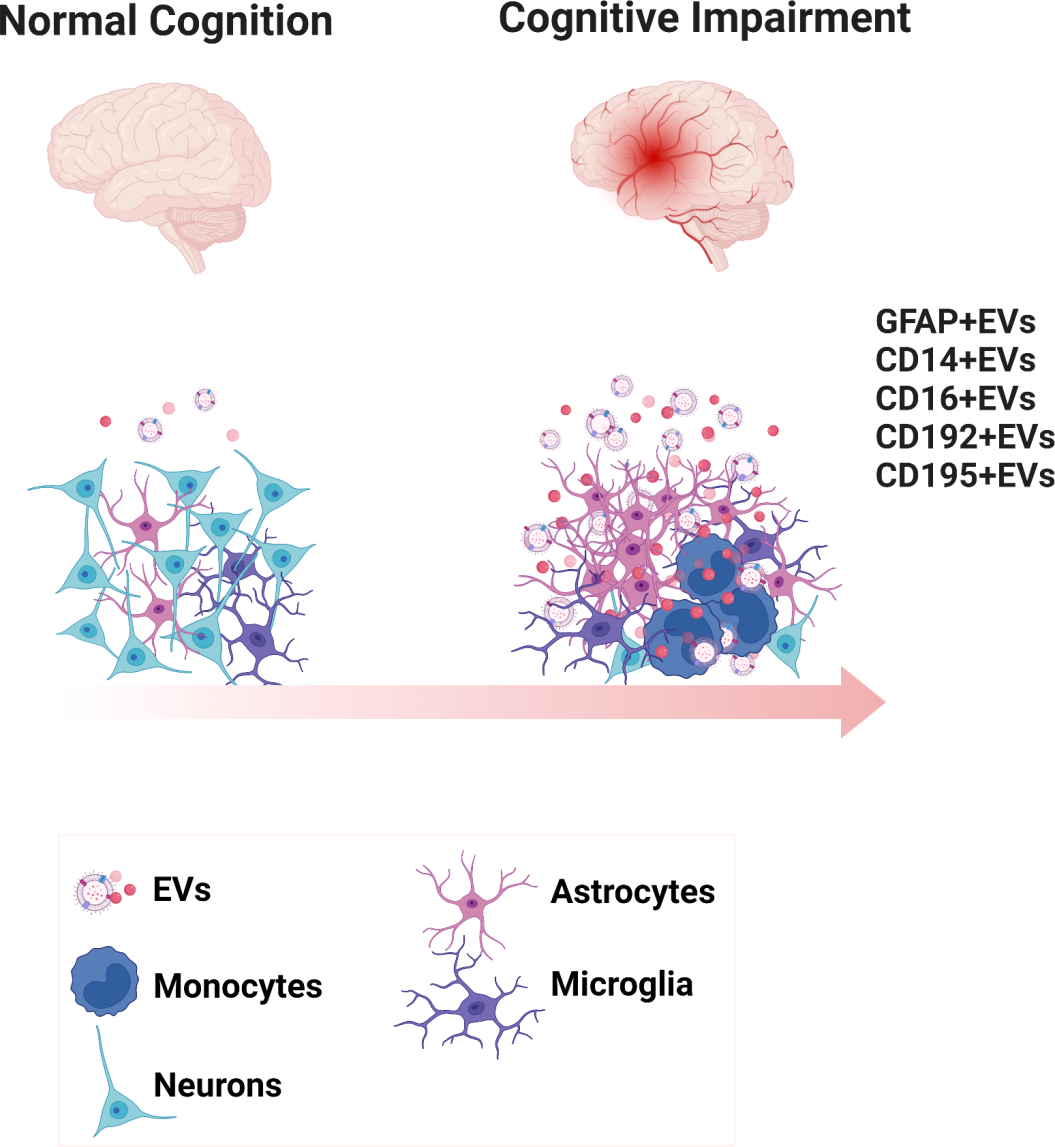
Diagrammatic representation of the biogenesis of EVs expressing inflammatory phenotypic markers in neurocognitive disorders. Scheme presents the healthy brain and the inflammatory environment of the cognitively impaired brain. EVs are generated reflecting the underlying surface proteins on their cell of origin.

To the best of our knowledge, this study is the first that provides evidence that neurocognitive impairment in HIV-infected individuals is associated with increased levels of both CSF and plasma EVs enriched with the neuronal marker GFAP compared to normal cognition. These results suggest that this neuronal damage biomarker in EVs may reflect brain pathological changes, and further studies would be needed to confirm if GFAP+EVs could represent a potential biomarker for HAND screening. This would be particularly valuable if a panel of biomarkers could be assembled that would be more predictive than each individual biomarker. Furthermore, we also found that plasma-EVs expressing monocyte-associate markers, including CD14, CD16, CD192, and CD195 were significantly elevated in HIV-infected individuals with cognitive impairment compared to those without cognitive impairment (**Figure 6**). We further revealed that circulating monocyte subsets were related not only with CSF and plasma EVs expressing monocytes and neuronal markers, but also with platelet markers in HIV-infected individuals with cognitive impairment. Together, these results indicate that EVs derived from both myeloid and neurological cell lineages may provide new insights into mechanisms impacting neurological disorders in treated HIV individuals.

Our findings are consistent with previously published data. In a longitudinal cohort, Flynn et al. reported that blood-derived EV concentrations of GFAP were higher in individuals with moderate and severe traumatic brain injury compared to controls (45). Further, Guha et al. reported that elevated levels of CSF-EVs correlated with the neuronal marker neurofilament light chain in treated HIV-infected individuals with neurocognitive impairment (46). A relationship between plasma neuron-derived EVs from HIV-infected individuals and CNS injury was also reported by Sun and colleagues, who investigated proteins (neurofilament-light chain, amyloid beta, and high mobility group box1) associated with neuronal damage (47). Furthermore, our group previously reported an association between neurocognitive impairment and a higher number of cells harboring HIV DNA selected from PBMCs enriched with CD14 monocytes (48). Notably, our findings predicted a higher number of monocyte-associated markers expressing EVs in HIV-infected individuals with cognitive impairment compared to those with normal cognition. Taken together, our findings and these observations suggest that higher abundance of EV-associated proteins related to neuroinflammation may reflect pathophysiological process in the CNS and both CSF and plasma EVs are a valuable source of new biomarkers.

Given the interactions of platelets with monocytes in HIV neurological disease (49, 50), the relationship found between EVs expressing the activated platelet marker CD62P and circulating inflammatory monocyte subsets in HAND subjects provides new insights into molecular mechanisms that could contribute to the development and progression of cognitive impairment. Consistent with our observations, previous reports have shown interactions of activated platelets and monocytes in the CNS contribute to HIV-associated neuroinflammation (49, 51, 52). Furthermore, consistent with this notion, Singh et al. showed that platelet-derived soluble CD40 ligand in the CSF and plasma from HAND individuals may contribute to blood-brain barrier permeability and neuroinflammation (49). As relevant examples, Furman et al. reported that monocyte/platelet aggregates in circulation were higher in subjects with myocardial dysfunction compared to healthy controls (53). Tian and colleagues reported that brain-derived EVs may promote platelet aggregation by binding to platelets and contribute to activation of the exogenous coagulation pathway and inflammatory response (54). However, very little is known about the effects of platelet-derived EVs in the pathological processes involved in HIV-associated neurological disorders. It is notable that using only a plasma sample we were able to identify correlates of neurological dysfunction that reflect the underlying cellular pathology. The ability to gain valuable information from plasma samples would vastly expand the number of individuals who can be studied, since banking PBMC samples is a costly and laborious process.

It is important to note that we observed a relationship with EV expressing neuroinflammatory damage markers (GFAP, MAP2, and CD200) and monocytes in the periphery in ART-treated HIV-infected individuals with cognitive impairment, suggesting the presence of a cross-talk between the periphery immune system and the brain during disease. Consistent with our observations, Farmen et al. reported a correlation between PBMC-derived monocytes with immune activation in the brain and changes of dopaminergic synapse function (55). Intermediate monocytes may play a prominent role, as other studies assessing monocyte subjects infected with HIV reported that intermediate monocytes could contribute to immune activation and inflammation (56–58), and could migrate across the blood-brain barrier (59). Another study performed by Veenhuis et al. reported that levels of intermediate monocytes are associated with worse cognitive function and may contribute to immune-brain interactions in HIV-infected individuals on virologically suppressive ART (60). A relevant study demonstrated that peripheral pro-inflammatory signals may be delivered to the CNS resident cells *via* choroid plexus-derived CSF EVs (61). Interestingly, studies have reported that EVs carry miRNA molecules that across the blood-brain barrier and may bind to activate surface or intracellular receptor, and this cross talk may lead to either pro- or anti-inflammatory responses (62–64). Based on our observations and published reports, our data suggest that EVs derived from myeloid immune process in HAND individuals may involve both periphery and brain and that both processes may be related. However, we propose that future studies should be focused on the mechanisms of how blood EVs-brain communicate with the CNS during systemic inflammation and elucidate the pathway targets that are affected by a portion of the candidate EV miRNAs in HAND-related neuroinflammation which could lead to new therapeutic strategies.

Our study has limitations; first, the study cohort consisted of HIV+ individuals with low CD4 nadir, and exposure to older ART regimens, so it is possible that some differences we found between cognitive impairment versus normal cognition could be confounded by factors other rather than cognitive status. Another limitation was that this cohort did not collect viral load in CSF samples, but no significant correlations were observed between circulating EVs and plasma viral load. Further investigations using a larger cohort and the use of a validation cohorts (with different clinical and demographic characteristics) of cognitive impairment in virally suppressed HIV-infected individuals are needed to confirm our findings. Finally, the progression of HAND is highly variable, and the molecular mechanisms underlying whether EVs and their cargo impact the resolution of inflammation to influence the accelerated progression of HAND in virally suppressed individuals remains undefined, though candidate mechanisms include EV-associated molecular cargo [reviewed in references (65, 66)]. Further investigation of these EVs to elucidate their potential as biomarkers of and monitor progression of disease and response to therapy in ART-treated HAND individuals is warranted.

## Data availability statement

The original contributions presented in the study are included in the article/**Supplementary Material**. Further inquiries can be directed to the corresponding author.

## Ethics statement

The studies involving human participants were reviewed and approved by the Institutional Review Board of the University of Hawaii Committee on Human Subjects. The patients/participants provided their written informed consent to participate in this study.

## Author contributions

EGMM designed and performed the experiments, analyzed data, and wrote the first draft of the article. JSL, SAB, LBG, and MLD analyzed data, CMS contributed clinical samples and edited the article, MAM analyzed data and edited the article, LCN contributed clinical samples, designed experiments, analyzed data, and edited the article, PJN designed experiments, analyzed data, and edited the article.

## Supplementary material

Supplementary Figure 1 Plasma-EVs expressing phenotypic markers in HIV-infected individuals on ART. Scatter plots of EVs/µL numbers (log 10-transformed). Plasma-EVs from HIV-infected individuals with cognitive impairment (CI) versus normal cognition (NC) according to the surface markers examined. There were no significant differences in the levels of EVs expressing the included markers using a two-tailed Mann-Whitney test.
