# Supplementary figures and images for: Circulating brain-derived extracellular vesicles expressing neuroinflammatory markers are associated with HIV-related neurocognitive impairment
Source: Front Immunol. 2022 Dec 19;13:1033712. doi: 10.3389/fimmu.2022.1033712 (PMC9806169; doi:10.3389/fimmu.2022.1033712)

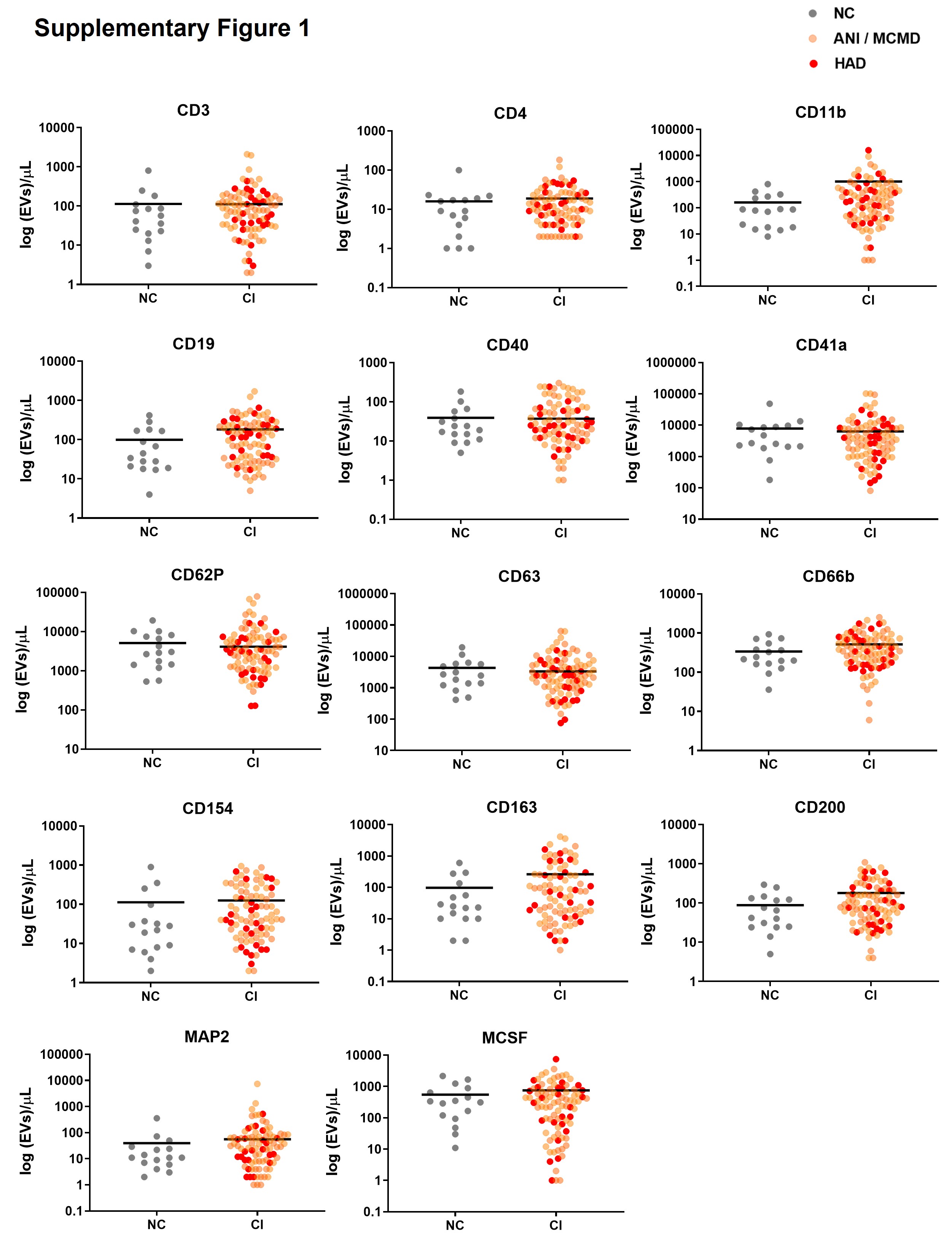

Supplement: Supplementary Figure 1 — Plasma-EVs expressing phenotypic markers in HIV-infected individuals on ART. Scatter plots of EVs/µL numbers (log 10-transformed). Plasma-EVs from HIV-infected individuals with cognitive impairment (CI) versus normal cognition (NC) according to the surface markers examined. There were no significant differences in the levels of EVs expressing the included markers using a two-tailed Mann-Whitney test. [file Image_1.tiff]
